# A new social gene in *Dictyostelium discoideum*, *chtB*

**DOI:** 10.1186/1471-2148-13-4

**Published:** 2013-01-09

**Authors:** Lorenzo A Santorelli, Adam Kuspa, Gad Shaulsky, David C Queller, Joan E Strassmann

**Affiliations:** 1 Department of Ecology and Evolutionary Biology, Rice University, Houston, TX 77005, USA; 2 Department of Molecular and Human Genetics, Baylor College of Medicine, Houston, TX 77030, USA; 3 Department of Biochemistry and Molecular Biology, Baylor College of Medicine, Houston, TX, 77030, USA; 4 Biology Department, Washington University in St. Louis, St. Louis, MO, 63130, USA; 5 Present address: Department of Zoology, University of Oxford, Oxford, OX1 3PS, UK

**Keywords:** Cheating behavior, Social evolution, *D*. *discoideum*, Pre-spore marker, *chtB*

## Abstract

**Background:**

Competitive social interactions are ubiquitous in nature, but their genetic basis is difficult to determine. Much can be learned from single gene knockouts in a eukaryote microbe. The mutants can be competed with the parent to discern the social impact of that specific gene. *Dictyostelium discoideum* is a social amoeba that exhibits cooperative behavior in the construction of a multicellular fruiting body. It is a good model organism to study the genetic basis of cooperation since it has a sequenced genome and it is amenable to genetic manipulation. When two strains of *D*. *discoideum* are mixed, a cheater strain can exploit its social partner by differentiating more spore than its fair share relative to stalk cells. Cheater strains can be generated in the lab or found in the wild and genetic analyses have shown that cheating behavior can be achieved through many pathways.

**Results:**

We have characterized the knockout mutant *chtB*, which was isolated from a screen for cheater mutants that were also able to form normal fruiting bodies on their own. When mixed in equal proportions with parental strain cells, *chtB* mutants contributed almost 60% of the total number of spores. To do so, *chtB* cells inhibit wild type cells from becoming spores, as indicated by counts and by the wild type cells’ reduced expression of the prespore gene, *cotB*. We found no obvious fitness costs (morphology, doubling time in liquid medium, spore production, and germination efficiency) associated with the cheating ability of the *chtB* knockout.

**Conclusions:**

In this study we describe a new gene in *D*. *discoideum*, *chtB*, which when knocked out inhibits the parental strain from producing spores. Moreover, under lab conditions, we did not detect any fitness costs associated with this behavior.

## Background

Microorganisms are able to communicate and cooperate to perform complex social behaviors once believed to be distinctive of multicellular organisms [1-5]. This includes formation of biofilms, foraging, spore dispersal and production of common goods. Cooperative groups are vulnerable to exploitation by cheaters, individuals that benefit from the product of cooperation without contributing their fair share [5-9]. In *Pseudomonas aeruginosa*[10] and *Myxococcus xanthus*[11,12], for example, the release of siderophores and digestive enzymes, respectively, represent costly activities that could be exploited by non-producers. In both organisms, strains grown in liquid culture for a number of generations sometime evolve to lose the ability to cooperate and instead behave as cheaters.

*Dictyostelium discoideum* displays cooperative behavior that provides a great model for the study of cheating. *D*. *discoideum* propagates as free-living unicellular amoebae feeding on bacteria associated with leaf litter, soil, and animal dung. In the past few decades, this social amoeba has been intensively studied because of its extraordinary development [13], which is a form of cooperation. When starving, cells aggregate and eventually form a multicellular organism capable of movement towards light and heat and away from chemicals such as ammonia [14,15]. Under the correct conditions, they develop into a fruiting body that represents the final stage of development [16]. About 20% of the cells differentiate into dead stalk cells that support the other 80%, which differentiate into viable spores. When spores are dispersed to a new source of food, they germinate to become amoebae and restart the vegetative stage of eating and dividing by binary fission. Stalk cells have been described as altruistic, since their death is presumed to aid the dispersal of the spores that form the next generation [17]. If the population consists of cells that are genetically identical, or related, it is possible to compare these individuals to somatic cells in a multicellular organism, or to a social insect colony. But when cells are genetically different, a conflict may arise over which cells survive and which form the stalk and die. In this case, a strain that differentiates more than its proportional share of spores is called a cheater and the other is called a loser.

In several cases, reproductive competition among cells within a chimera has been reported, but the cheater strains often carry a fitness cost relative to non-cheaters even to the extent that they are incapable of sporulating efficiently on their own [18-20]. On the other hand, in *D*. *discoideum* several strains isolated from the wild are capable of cheating and of producing fruiting bodies independent of other clones [17]. A large number of knockout or reduced function mutations generated in the laboratory also show such facultative cheating behavior [21]. These mutants are capable of producing spores in pure populations, but preferentially become spores and not stalk cells when mixed with the ancestral strain.

In *D*. *discoideum*, many genes have been reported to be involved in the disruption of cooperation, but not much is known about the underlying genetic mechanisms. The analyses performed so far on cheater mutants showed that multiple mechanisms and pathways may be involved in cheating behavior, with cheater mutants showing diverse functions including GTPase regulatory activity, polyketide synthesis, nucleotide binding, and phosphoric ester hydrolase activity [21]. Some of these pathways are involved in cell-cell communication, formation of secondary metabolites, or signal transduction. They can affect the communication pathway that regulates the proportion of spores and stalk cells [22], or disrupt the ubiquitin-ligase pathway responsible for the breakdown of certain target proteins [19]. Other mechanisms that can lead to cheating [23] might be the expression of spore genes earlier than prestalk genes [24]; premature entrance into development; presence of multinucleated cells that lead to more cell divisions during development; cannibalism, as is present in *Dictyostelium caveatum*[25]; and production of a killer factor as reported in *Polysphondylium pallidum*[26].

Cheater strains in the Dictyostelidae typically have a fitness cost or some deleterious pleiotropic effect that prevents the mutant from spreading, since otherwise a loss of function mutant would easily evolve. This is the case of mutants *chtA*, *dimA*, and *csaA*[19,27,28]. In this work we characterize a mutant called *chtB* that can facultatively cheat when mixed with the parental strain, but does not suffer from an obvious fitness cost in the laboratory. When mixed with *chtB*, the parental strain produces fewer spores and expresses a lower level of the pre-spore gene *cotB*.

## Results

A mutant called *chtB* (cheater B) was recovered at the end of a selection for mutants that preferentially produce spores rather than stalks in a mixed population [21].

In the parental strain AX4, the chtB transcript appears early in development and is completely absent in *chtB* mutant cells. This mutant produces a higher number of spores than AX4 in chimeras that are made with equal numbers of cells of the two strains. When it is plated clonally it shows a normal developmental phenotype, so it is not dependent on parental cells in a social stage chimera. The loss of function of the *chtB* gene also increases expression of *cotB*, a prespore marker, early in development, indicating the mechanism of action is early specialization as spore over the ancestral strain. On the other hand, parental cells reduce the expression of *cotB* and differentiate a lower number of spores in chimera. We tested *chtB* mutants to detect whether fitness costs were associated with its cheating ability. Not only did we not detect any fitness cost, but we also found that the mutant presents the same sporulation efficiency and faster doubling time when grown in liquid, than AX4. Finally, the presence of *chtB* mutant cells in chimera with parental strain limits the expression of a pre-spore marker in the latter. As a consequence the parental strain is unable to produce its fair share of spores.

### Isolation of the *chtB* mutants

**Figure 1:**
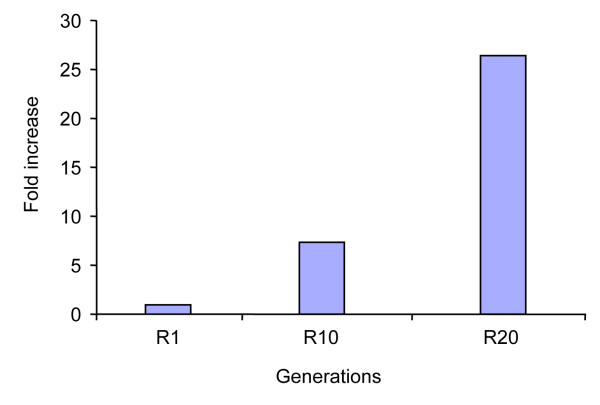
**The *chtB *mutant increases in frequency during the selection.** Q-PCR using DNA from the entire population at the 1^st^, 10^th^ and 20^th^ generation (R1, R10 and R20) and using primers specific to the *chtB* allele, shows that the mutant *chtB* increased 26.4 fold by the end of the selection.

We isolated the *chtB* mutant during a selection for cheaters from a pool of 10,000 mutants [21]. This pool of mutants was subjected to 20 rounds of growth, development, and spore germination in a mixed population so that cheaters that differentiate into spores with a higher efficiency would become enriched in the evolving population. At the end of the selection, *chtB* was one of the mutants that were randomly chosen to be tested for cheating properties. To confirm that *chtB* really increased its frequency during the selection, we used quantitative PCR (Q-PCR) to obtain information about the abundance of the *chtB* allele. We extracted genomic DNA from the entire population after the 1^st^, 10^th^, and 20^th^ generations of the selection and used gene-specific primers to quantify the *chtB* allele. The mutant *chtB* increased 7.4 fold at the 10^th^ generation and 26.4 fold at the 20^th^ (Figure 1), thus supporting the hypothesis that this cheater increased in frequency during the selection.

### The *chtB* gene

**Figure 2:**
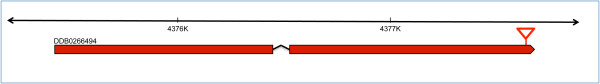
**The *chtB *gene is located on the chromosome 5.** The gene contains one short intron (thin black line) and two exons (thick red lines) as indicated in the gene model. The red triangle indicates the position of the insertional mutation generated by REMI.

The mutant *chtB* was generated by insertional mutation of the pBSR1 plasmid [29] in chromosome 5 at position 4377789 towards the 3’ end of the ORF of the gene *DDB*_*G0290617* (Figure 2). The predicted protein has not been studied before and it consists of a FNIP repeat (named FNIP after the pattern of a conserved motif found only in *D*. *discoideum*). We named the gene *chtB* because its social behavior resembles the previously described *chtA* mutant [19]. To verify that the insertion was responsible for the mutant phenotype, the mutation was recapitulated by homologous recombination in the AX4 genetic background using the rescued plasmid as a knockout vector [30]. We confirmed the mutation by Southern blot hybridization using a gene-specific probe and tested the strain for cheating.

**Figure 3:**
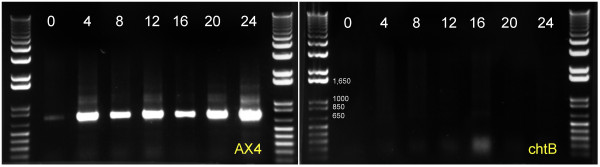
**The *chtB *gene is expressed during development and is completely silent in our mutant.** RT-PCR reaction using RNA extracted during a time course (time points indicated in hours) shows that the *chtB* mRNA is observed only in AX4 during development. No mRNA is detected in the mutant *chtB.*

RT-PCR analysis showed that the parental strain AX4 expressed the *chtB* mRNA at 0 hours of development and mRNA abundance was greatly increased at 4–24 hours (Figure 3). In the mutant cells, *chtB* mRNA was not expressed at any time.

### The *chtB* mutant cheats on the parental strain

**Figure 4:**
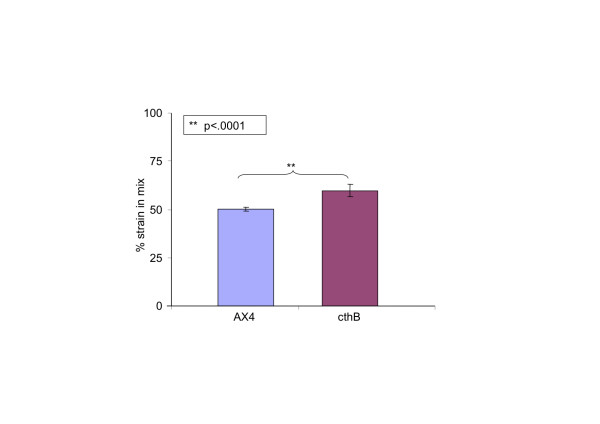
**The *chtB *mutant cheats on AX4.** When mixed with the parental strain AX4 [act15/GFP], *chtB* differentiates 59.9±3.3% (n=22) of the total spores. Control AX4 cells mixed with AX4 [*act15*/GFP] cells differentiate 50.1±1.0% (n=27) of the total number of spores. The *chtB* strain differentiates a significantly higher number of spores than the parental strain (Two tailed t-test, p<.0001. Bars represent standard errors).

To be defined as a cheater, the mutant *chtB* must produce significantly more than 50% of the spores in a pairwise mixing experiment with the parental strain, and this was the case. When mixed at equal proportions with AX4, *chtB* differentiated 59.9±3.3% of the total number of spores. This result differed significantly from the control chimera between AX4 and AX4 [act15/GFP] (p<.0001, T-test; Figure 4).

### Analysis of fitness cost associated with cheating

#### *chtB* has no overt morphological defects

**Figure 5:**
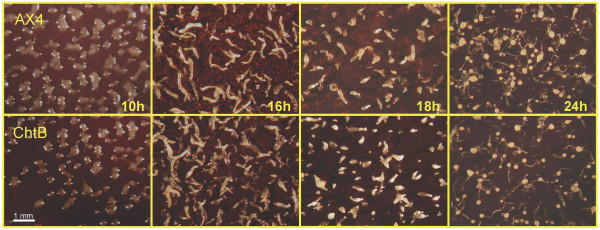
**Developmental morphologies of AX4 and *chtB*.** We grew the cells in axenic medium and developed them separately, as indicated, on nitrocellulose filters. We photographed the cells from above at the indicated times (bar = 1mm).

To test whether *chtB* shows any morphological difference relative to its ancestor, we observed cells of both strains at different stages of development. In both parent and knockout, we observed loose aggregates at about 10 hours, tight aggregates at 12 hours, fingers at 16 hours and Mexican hats at 20 hours. Between 20 and 24 hours both strains culminated, leading to the formation of well-proportioned fruiting bodies consisting of stalks cells and spores (Figure 5). We saw no noticeable differences between the phenotypes of AX4 and *chtB* developed on filters.

#### *chtB* differentiates a similar number of spores than AX4 in a pure population

A fitness cost for a cheater could be manifested as reduced sporulation when developing in a pure population. To test that possibility we developed *chtB* cells in a pure population or mixed with AX4 cells. The results (see Additional file 1: Figure S1) show that the sporulation efficiency of *chtB* (83.11±4.69%, n=10) is not significantly different from AX4 (71.53±5.82%, n=10, t-test p<0.14), suggesting that the mutation does not have a sporulation-related fitness cost. The chimera sporulation efficiency was similar to both pure populations (84.16±7.26%, n=10, t-test p=0.19 vs. AX4, p=0.90 vs. *chtB*).

#### *chtB* spores are viable

We also tested whether a fitness cost may be associated with the spore germination efficiency. Our results show that *chtB* was able to germinate a number of spores (72.3±16.2%) comparable to AX4 (75.5±15.1%, t-test, p=0.08, n=6). This result indicates that *chtB* does not produce fewer viable spores than its parent (Additional file 2: Figure S2).

#### *chtB* grows faster in liquid medium

**Figure 6:**
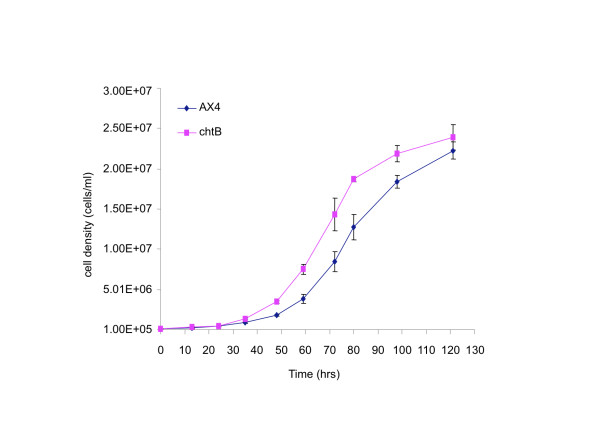
**Growth curves of AX4 and *chtB *cells in liquid HL5 medium.** Cell densities of the two strains, as indicated, are plotted as a function of time (hrs).

To compare the growth rates of the *chtB* mutant and its ancestor, we grew cultures of *chtB* and AX4 in liquid media starting at a cell density of 1x10^5^ cells/ml. *chtB* reached log phase after about 40 hours, while AX4 took about 10 hours longer. When in logarithmic phase, *chtB* cells showed a doubling time of 7.6±0.7 hours (Figure 6) while AX4 cells showed a doubling time of 10.3±1.5 hours (t-test, p<0.05, n=3), showing that *chtB* has a faster doubling time than AX4 in liquid medium.

#### Alteration of cell type proportioning as cheating mechanism

We assessed cell-type proportioning by measuring beta-galactosidase activity in cells that express the marker under the promoters of either *cotB*[31] (a prespore marker) or *ecmA*[32] (a prestalk marker). We measured the overall enzyme activity in the population using an ONPG-assay and the number of cells of each type using X-gal staining as described [33]. We compared the level of *lacZ* expression in chimerae between the reporter strains and AX4 or *chtB* to determine the effect of *chtB* on prespore and prestalk differentiation in the AX4 victim.

#### Prespore differentiation

**Figure 7:**
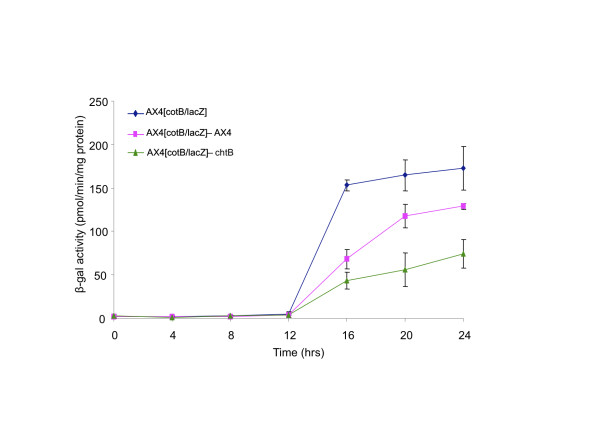
**ONPG analysis of AX4 cells expressing the *lacZ *gene under the prespore promoter *cotB*.** Cells were developed alone or mixed at a 1:1 ratio with AX4 and *chtB* cells. Developmental time points (hours) are represented on the x-axis and beta-galactosidase activity on the y-axis. When AX4 [*cotB*/*lacZ*] are mixed with *chtB* cells, the β-galactosidase activity is lower than when mixed with AX4 (16, 20 and 24 hour time points, t-test, p<0.05, n=3).

ONPG analysis of AX4 [*cotB*/*lacZ*] showed that *cotB* is not expressed until 12 hours. Then it starts increasing and reaches saturation at 16 hours (Figure 7). If AX4 [*cotB*/*lacZ*] cells were mixed at a 1:1 ratio with AX4 cells that do not express *lacZ*, the level of β-galactosidase activity is about half of that produced by AX4 [*cotB*/*lacZ*] alone. When AX4 [*cotB*/*lacZ*] cells were mixed at the same ratio with *chtB* cells, the β-galactosidase activity was significantly lower than in the mix with AX4 (16, 20 and 24 hour time points, t-test, p<0.05, n=3). These results show that in chimeras *chtB* is able to reduce the expression of *cotB* in the wild type cells and that AX4 cells are forced to form fewer prespore-cells than their fair share.

#### Prestalk differentiation

**Figure 8:**
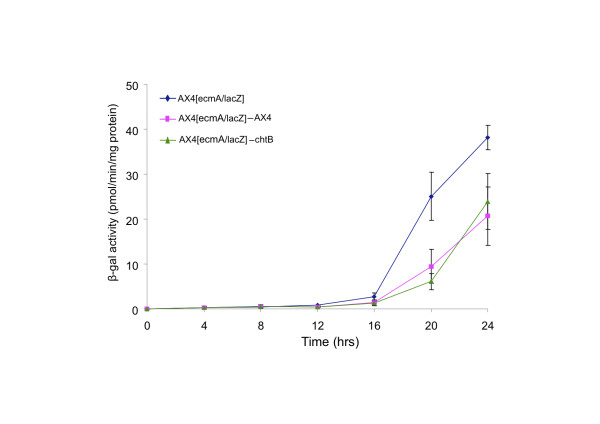
**ONPG analysis of AX4 cells expressing the *lacZ *gene under the prestalk promoter *ecmA.*** Cells were developed alone or mixed at a 1:1 ratio with AX4 and *chtB* cells. Developmental time points (hours) are represented on the x-axis and beta-galactosidase activity on the y-axis. β-galactosidase activity did no differ when AX4[*ecmA*/*lacZ*] cells are mixed with AX4 or with *chtB.*

To test whether the presence of *chtB* cells in chimeras affects prestalk cell formation in the victim, we performed a similar analysis using the strain AX4[*ecmA*/*lacZ*] alone and in pairwise mixes with AX4 and *chtB* (Figure 8). In this case, no significant differences were seen in the β-galactosidase activity when AX4[*ecmA*/*lacZ*] cells are mixed with AX4 or with *chtB*. We conclude that the presence of *chtB* cells in chimeras does not influence the expression of the prestalk marker *ecmA* in the wild type cells.

#### Developmental cell fate

**Figure 9:**
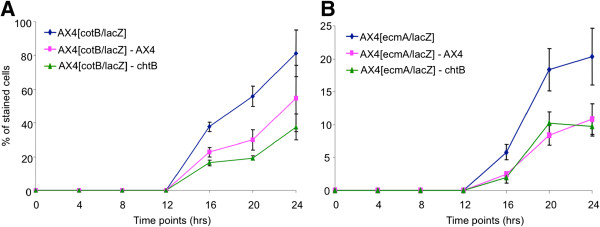
**Changes in the proportion of prespore cells but not in prestalk cells.** (**A**) Cells expressing *lacZ* under the *cotB* promoter or (**B**) under the *ecmA* promoter were developed alone or mixed with AX4 or *chtB* in a 1:1 ratio. Developmental time points (hours) are represented on the x-axis and percentage of stained cells on the y-axis. AX4[*cotB*/*lacZ*] mixed with *chtB* at the 16 and 20 hours time point, shows a significantly lower percentage of stained cells than when mixed with AX4 (for the 16 and 20 hours time points, t-test, p<0.05, n=3). AX4[*ecmA*/*lacZ*] mixed with *chtB* does not show a significant difference in the percentage of stained cells than when mixed with AX4. (For 16, 20 and 24 hour time points, t-test, p>0.26, n=3).

The results obtained with ONPG analysis show that the presence of chtB cells in a mix with AX4 reduces the promoter activity of the pre-spore gene cotB. This observation could be due to reduced cotB expression in all the wild type prespore cells, or to a reduction in the number of prespore cells in the wild type. We addressed this issue by counting the number of cells that expressed the marker gene. When AX4 [cotB/lacZ] were mixed in equal proportions with chtB (Figure 9A) they produced a significantly lower percentage of stained cells than when mixed with AX4 (for 16 and 20 hour time points, t-test, p<0.05, n=3). Therefore we conclude that, in chimera with wild type, chtB forces the parental cells to reduce the proportion of prespore cells and, as a consequence, to produce fewer spores. When the same test was performed using the AX4 [ecmA/lacZ] strain (Figure 9B), there was no difference between the number of stained cells observed in mixes of AX4[ecmA/lacZ] with AX4 or chtB (for 16, 20 and 24 hours time points, t-test, p>0.26, n=3). We conclude that the presence of chtB in chimera did not affect the number of prestalk A cells produced by the victim strain, but could have made them become prestalk B (pstB) cells, which normally produces the basal disc of the fruiting body, or pstO cells, which occupy the rear half of the prestalk region.

## Discussion

We isolated and characterized a mutant called *chtB* that cheats without overt fitness costs. *chtB* was isolated after a genetic selection for cheaters that were able to form fruiting bodies clonally [21].

The mechanism by which *chtB* cheats in chimeras is novel. *chtB* reduced the expression of the prespore marker *cotB* in the wild type population as a consequence of the wild type strain forming fewer prespore cells than it does in control mixtures. One might expect then that these missing wild type prespore cells end up making stalk, but we found no evidence for this; *chtB* does not alter the wild type expression of the prestalk marker *ecmA*. It is therefore unclear what happens to the wild type cells that would have produced spores.

To test whether the *chtB* mutation was associated with fitness costs, we tested the strain’s growth, developmental morphology, sporulation and germination efficiency and found no adverse effects compared to the ancestor AX4. Indeed there was some evidence of better performance by the mutant. The *chtB* mutant grows faster than the wild type in liquid medium. This finding cannot account for the increase in frequency of *chtB* during the original selection, because the selection was performed on agar plates, where *chtB* does not have a growth advantage [21].

The behavior of the mutant raises both mechanistic and evolutionary questions. On the mechanistic side, we begin by asking whether its higher representation in spores is due to either fixed or facultative allocation strategies [34]. In the fixed allocation strategy, the cheater produces more spores than the loser when each are grown as pure clones. In this case, the cheater has not changed its behavior in chimera; its solitary strategy carried over to the chimeric setting gives it an advantage. In the facultative allocation strategy, the cheater produces more spores in chimeras than expected from its solitary production. The spore production of *chtB* is not significantly higher than that of AX4, suggesting it is a facultative cheater. However the point estimate is higher, so it is worth considering whether, if that non-significant difference were real, it could explain cheating as a fixed strategy. In fact it cannot, as the 59.9% representation observed in chimeras is significantly more than the expected fixed allocation of 54.0% (calculated from *chtB* on its own divided by the sum of *chtB* on its own and wildtype on its own, or 83.1/(71.5 + 83.1)). Thus *chtB* appears to be a facultative cheater, indicating that an additional cheating mechanism is at play besides fixed higher spore production. Several wild clones of *Dictyostelium discoideum*[17] and many laboratory mutants [21,22] are also cheaters that do not exhibit overt phenotypic abnormalities, but the previous studies did not explore possible costs that might be associated with the cheating behavior. Cheater strains in a population can be viewed as parasites since they use common resources without paying the cost, which could lead to the collapse of the entire social machinery. Several mechanisms have been suggested to explain why cheaters do not take over the entire population [35]. High relatedness is an important control of cheaters, because it means that like groups with like, so cheaters have reduced opportunity to exploit cooperators [36,37]. This explanation does not seem to apply to *chtB* because it does at least as well as wild type even when alone, so it would appear to be favored even at high relatedness, with an added boost from cheating in any chimeric mixtures.

With or without high relatedness, disadvantageous pleiotropic effects can also keep a mutant from spreading [27]. We detected no pleiotropic disadvantages of *chtB*. However, there are two important caveats. First, all the cheating experiments reported so far were performed in the laboratory under controlled environments. In the wild, however, factors like temperature, pH, and moisture could influence both cheating efficiency and pleiotropic effects. The *chtB* knockout behaves as a cheater under the conditions that we have used, but it may not be able to cheat in nature. Likewise, inactivation of *chtB* had no apparent adverse consequences in the laboratory, but it must have a cost that would limit its propagation in nature, or the gene would have been lost. Second, selective forces too small to effectively be detected in the laboratory could still be important in nature. One hypothesis that combines these two caveats is that *chtB* might suffer from reduced dispersal from shorter stalks. If *chtB* produces more spores, it may produce shorter stalks. We did not attempt stalk measurements because they are far less accurate and more variable than spore measurements. Shorter stalks might reduce dispersal, presumably by animal vectors. Another possibility is that the mechanism by which *chtB* knockouts achieve their success in chimera involves earlier fruiting. If this is the case, it could be that this is normally a disadvantage, keeping the mutant from spreading.

## Conclusion

In this study we describe a novel mechanism by which a *D*. *discoideum* cheater exploits parental strain cells by inhibiting them from producing spores. One way to understand how cooperative behaviours have evolved and are maintained is to identify the mechanisms cheaters use to exploit such cooperation. Moreover, for the first time we concluded that under our experimental conditions there are no fitness costs associated with the cheater trait investigated, unlike the disadvantageous pleiotropic effects that are suggested mechanisms preventing cheaters from spreading in a population.

## Methods

### Strains and growth conditions

The *Dictyostelium discoideum* strains used were the axenic strain AX4 [38], AX4 [actin15/GFP], and the REMI mutant *chtB*[21]. Cells were grown in suspension in HL-5 medium or on SM agar plates in association with *Klebsiella aerogenes*[39]. HL-5 was supplemented with the antibiotics G418 or Blasticidin S (both at 5μg/ml), as required.

### Increase in frequency of the *chtB* gene during the screen

Quantitative PCR was performed using the DNA Opticon® Engine system BIO-RAD [40]. As template for the reaction we used genomic DNA extracted from the 1^st^, 10^th^ and 20^th^ round of selection. Specific *chtB* primers were used to obtain amplification. IG7 (a constitutively expressed gene) was used as the loading control. SYBR Green was used as the dsDNA fluorescent dye.

### RNA extraction

AX4 and *chtB* cells were plated on two black filters each (5x10^7^ cells/filter) and the entire populations were collected at different developmental time points. We extracted RNA using the TRIzol reagent (Invitrogen) according to the manufacturer’s recommended procedures and dissolved it in 100 μl of 1X MOPS buffer [41].

### *chtB* gene expression

Total RNA was reverse transcribed using SuperScript™ II Reverse Transcriptase kit (Invitrogen) and oligo d(T) primers. Q-PCR was performed on the resulting cDNA using *chtB* gene-specific primers. The products were resolved by electrophoresis on a 0.8% agarose gel, stained with ethidium bromide, and observed under UV light.

### Cheating assays

The strain *chtB* was tested for cheating behavior in pairwise mixing experiments using the GFP-labeled strain method as described [21].

### Developmental morphology

Wild type and mutant cells were grown in HL5 liquid medium. Once in log phase (between 1×10^6^ and 5×10^6^ cells/ml), cells were washed twice with KK2 buffer (16.3mM K_2_HPO_4_, 3.7mM KH_2_PO_4_, pH 6.2) and resuspended in PDF (20mM KCl, 5mM MgCl_2_, 9mM K_2_HPO_4_, 13mM KH_2_PO_4_, 0.3mM streptomycin sulfate, pH 6.4) at a density of 5×10^7^ cells/ml. 1 ml of this cell suspension was deposited on Black nitrocellulose filters [38] and incubated at 22°C. Pictures were taken every two hours.

### Sporulation efficiency

We plated 5x10^7^ cells on a KK2 agar plate both clonally and in 1:1 ratio. After 30 hours the entire contents of the plates were collected and resuspended in 1 ml of KK2 with 0.1% NP40 so that only spores could survive. To assess spore number, a measured aliquot from each sample was counted with a haematocytometer. The sporulation efficiency was calculated by dividing the number of spores collected at the end of development by the total number of cells plated initially.

### Germination efficiency

Spores were counted and plated out at low density (100–500 per plate) on SM plates with 300 μl of *Klebsiella aerogenes*. After a few days, single plaques were observed in the bacterial lawn, each representing a viable spore. The proportion between the number of plaques observed and the number of the spores plated indicated the germination efficiency.

### Doubling time

Cells were inoculated at a density of 1×10^5^ cells/ml in 250ml Pyrex flasks containing 50 ml of HL5. The flasks were shaken at 200 rpm at 22°C until the cultures reached stationary phase at a density of about 2.5 × 10^7^cells/ml. Samples were collected at 12 hour intervals and the cells were counted. Each experiment was repeated three times.

### ONPG (ortho-Nitrophenyl-β-galactoside) analysis and X-gal staining

The strains used for this analysis were TL1 (AX4 [*cotB*/*lacZ*]), TL6 (AX4 [*ecmA*/*lacZ*]), the *chtB* mutant and the parental strain AX4. Strains expressing the *lacZ* gene were grown in the presence of 5μg/ml G418. Cells were grown in HL5 liquid medium and then plated on black filters as described previously. Each *lacZ* strain was plated alone or mixed in 1:1 ratio with AX4 and *chtB*. AX4 [*cotB*/*lacZ*] cells were plated in a pure population or mixed at a 1:1 ratio with AX4 and *chtB* on white nitrocellulose filters.

### ONPG analysis

The contents of each filter was washed in KK2 and resuspended in 1ml of Z buffer with 1% Triton in order to extract the protein content. For each sample 5 μl were used to determine the protein concentration with a Bradford assay [42]. After normalizing the protein concentration of all the samples, 200 μl of the protein extract was added to 200 μl of Z buffer and 200 μl of ONPG solution (4 mg ortho-nitrophenyl-b-D-galactopyranoside in 1ml of 0.1M Z buffer). This mixture was incubated at room temperature and gently shaken until the yellow color developed to the desired intensity. The reaction was stopped by adding 400 μl of 1M Na_2_CO_3_ and the time recorded. The absorbance of the solution was measured with a spectrophotometer at 420 nm wavelength. β-galactosidase activity was calculated using the following formula:

$$\begin{array}{l} \text{Specific}\;\beta -\text{gal}.\text{activity}\\ \;=({\text{A}}_{420}\times 2\times 10^{6})/(4700({\text{A}}_{420}/\text{mol ONPG})\\ \;\times {\text{protein}}^{\text{mg}}{/}_{\text{ml}}\times \text{vol}(\text{ml})\times \text{time}\;(\text{min})) \end{array}$$

### X-Gal staining of cells in suspension

Cells were collected from filters at different time points, resuspended in Pronase buffer (0.1% Pronase, 14mM β-mercaptoethanol, 150mM NaCl, 50mM Tris, pH 7.0) and dissociated by trituration. The cells were fixed stained with X-gal as described [43] and counted under a microscope. For each data point we counted between 150 to 300 cells and determine the percentage of stained cells over the total number.

## Competing interests

The authors declare that they have no competing interests.

## Authors’ contributions

Conceived and designed the experiment and advised on experimental procedure, analysis and writing: LAS, AK, GS, DCQ and JES. Performed the experiments LAS; analyzed the data and wrote the paper: LAS, AK, GS, DCQ, and JES. All authors read and approved the final manuscript.

## Supplementary Material

Additional file 1: Figure S1 Spore production in pure populations and in chimeras. In each experiment we plated 5x10^7^ cells on a KK2 plate and allowed them to develop. After 24 hours the contents of the plates were collected, treated with detergent and the spores counted. The sporulation efficiency of *chtB* is not significantly different from AX4. Click here for file

Additional file 2: Figure S2 Germination efficiency assay. Spores of *chtB* and AX4 strains were plated on SM plates in association with bacteria and the number of viable spores was inferred from the number of plaques formed. *chtB* produce a percentage of viable spores comparable to AX4. Click here for file
