# Supplementary figures and images for: A new social gene in Dictyostelium discoideum, chtB
Source: BMC Evol Biol. 2013 Jan 9;13:4. doi: 10.1186/1471-2148-13-4 (PMC3559258; doi:10.1186/1471-2148-13-4)

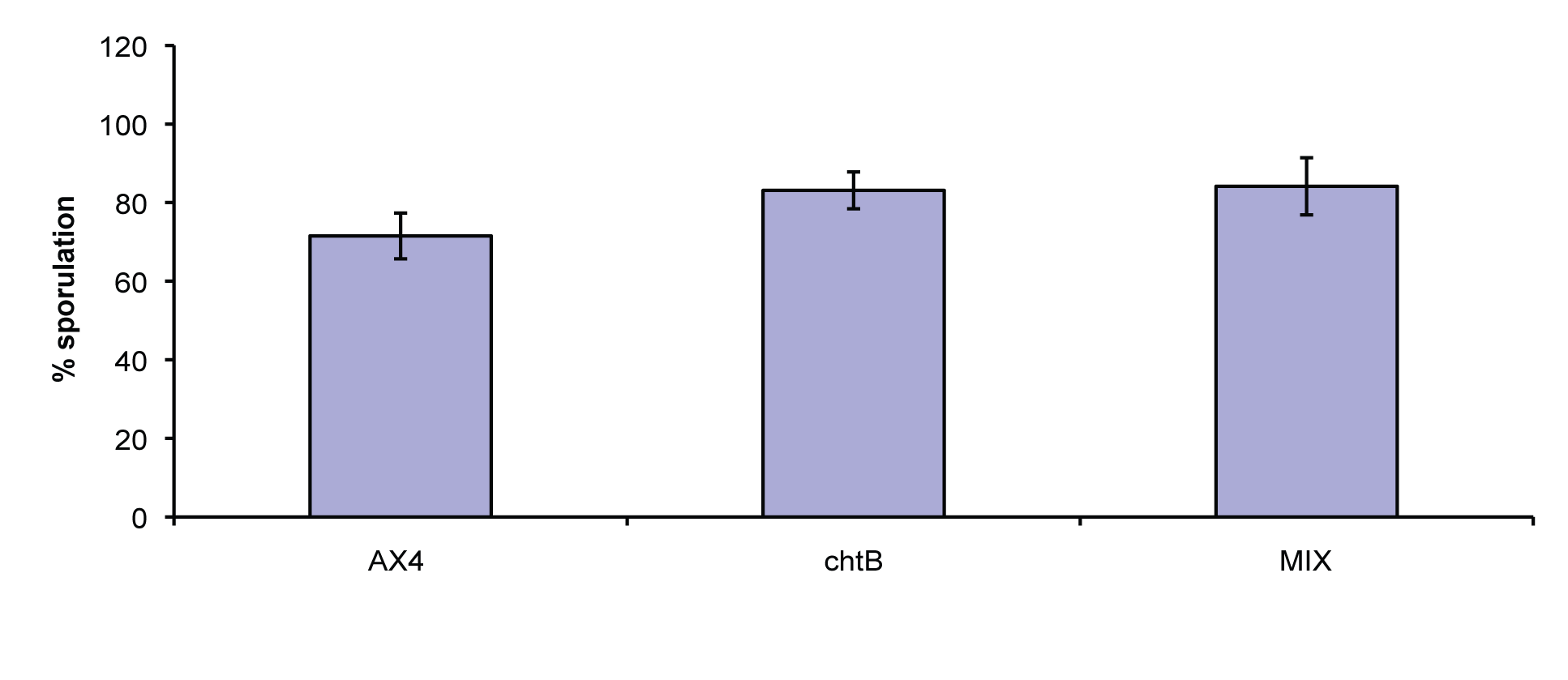

Supplement: Additional file 1: Figure S1 — Spore production in pure populations and in chimeras. In each experiment we plated 5x10^7^ cells on a KK2 plate and allowed them to develop. After 24 hours the contents of the plates were collected, treated with detergent and the spores counted. The sporulation efficiency of chtB is not significantly different from AX4. [file 1471-2148-13-4-S1.tiff]

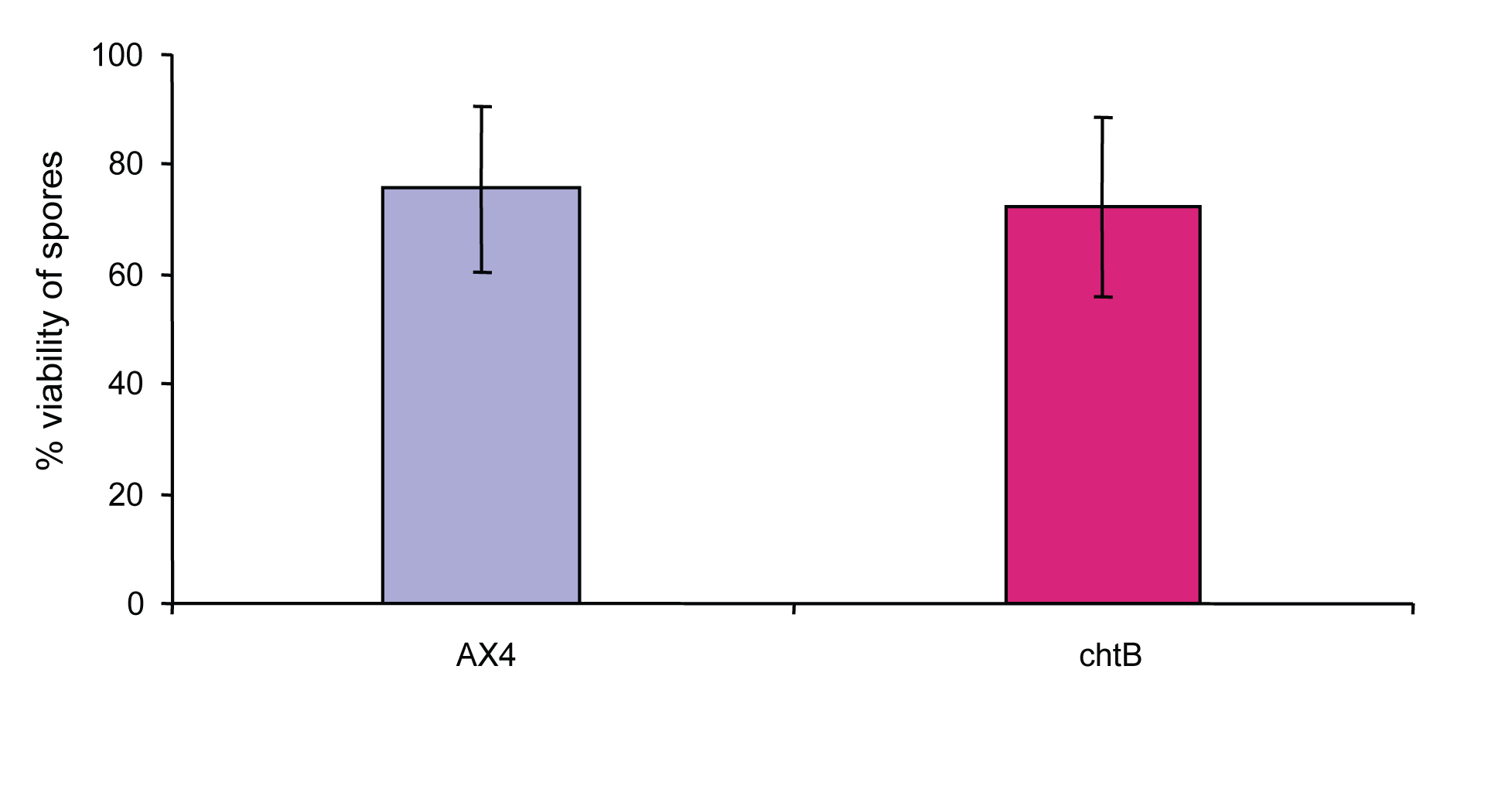

Supplement: Additional file 2: Figure S2 — Germination efficiency assay. Spores of chtB and AX4 strains were plated on SM plates in association with bacteria and the number of viable spores was inferred from the number of plaques formed. chtB produce a percentage of viable spores comparable to AX4. [file 1471-2148-13-4-S2.tiff]
